# Recurrent rearrangements of the Myb/SANT-like DNA-binding domain containing 3 gene (*MSANTD3*) in salivary gland acinic cell carcinoma

**DOI:** 10.1371/journal.pone.0171265

**Published:** 2017-02-17

**Authors:** Nicholas Barasch, Xue Gong, Kevin A. Kwei, Sushama Varma, Jewison Biscocho, Kunbin Qu, Nan Xiao, Joseph S. Lipsick, Robert J. Pelham, Robert B. West, Jonathan R. Pollack

**Affiliations:** 1 Department of Pathology, Stanford University School of Medicine, Stanford, California, United States of America; 2 Genomic Health, Redwood City, California, United States of America; 3 Arthur A. Dugoni School of Dentistry, University of the Pacific, San Francisco, California, United States of America; University of Michigan, UNITED STATES

## Abstract

Pathogenic gene fusions have been identified in several histologic types of salivary gland neoplasia, but not previously in acinic cell carcinoma (AcCC). To discover novel gene fusions, we performed whole-transcriptome sequencing surveys of three AcCC archival cases. In one specimen we identified a novel *HTN3*-*MSANTD3* gene fusion, and in another a novel *PRB3*-*ZNF217* gene fusion. The structure of both fusions was consistent with the promoter of the 5’ partner (*HTN3* or *PRB3*), both highly expressed salivary gland genes, driving overexpression of full-length MSANTD3 or ZNF217. By fluorescence *in situ* hybridization of an expanded AcCC case series, we observed *MSANTD3* rearrangements altogether in 3 of 20 evaluable cases (15%), but found no additional *ZNF217* rearrangements. *MSANTD3* encodes a previously uncharacterized Myb/SANT domain-containing protein. Immunohistochemical staining demonstrated diffuse nuclear MSANTD3 expression in 8 of 27 AcCC cases (30%), including the three cases with *MSANTD3* rearrangement. MSANTD3 displayed heterogeneous expression in normal salivary ductal epithelium, as well as among other histologic types of salivary gland cancer though without evidence of translocation. In a broader survey, MSANTD3 showed variable expression across a wide range of normal and neoplastic human tissue specimens. In preliminary functional studies, engineered MSANTD3 overexpression in rodent salivary gland epithelial cells did not enhance cell proliferation, but led to significant upregulation of gene sets involved in protein synthesis. Our findings newly identify *MSANTD3* rearrangement as a recurrent event in salivary gland AcCC, providing new insight into disease pathogenesis, and identifying a putative novel human oncogene.

## Introduction

In cancers, chromosomal translocations and rearrangements can create gene fusions, resulting in the effective overexpression of full-length cancer genes or producing chimeric oncoproteins [1, 2]. Gene fusions were originally considered hallmarks of hematopoietic and mesenchymal cancers, but more recently have been reported in a wide range of epithelial cancer types [2]. Identifying and characterizing gene fusions have provided new molecular insight into disease pathogenesis. Moreover, gene fusions have refined the diagnosis and classification of cancers, and have proven to be effective targets for molecularly-directed therapy. Examples include the classic *BCR*-*ABL* fusion in chronic myelogenous leukemia [3], and more recently, *EML4*-*ALK* in lung cancer [4].

Salivary gland neoplasms comprise a collection of at least 30 distinct histopathologic subtypes, including malignant epithelial tumors (e.g. acinic cell carcinoma, adenoid cystic carcinoma, mucoepidermoid carcinoma, and salivary duct carcinoma) and benign epithelial lesions (e.g. pleomorphic adenoma, basal cell adenoma, oncocytoma, myoepithelioma, and Warthin tumor). Developing mainly in the parotid gland, these diagnoses together account for approximately 6% of all head and neck tumors [5]. Over the past years, recurrent gene fusions have been identified in several different salivary gland neoplasms [6–8], including *PLAG1* fusions (most often *CTNNB1*-*PLAG1*) and *HMGA2* rearrangements in pleomorphic adenoma, *CRTC1*-*MAML2* fusion in mucoepidermoid carcinoma, *MYB*-*NFIB* (or *MYBL1*-*NFIB* [9, 10]) fusion in adenoid cystic carcinoma, *ETV6*-*NTRK3* fusion in mammary analogue secretory carcinoma of the salivary gland, and *EWSR1*-*ATF1* fusion in hyalinizing clear cell carcinoma. These fusions, impacting cell signaling pathways and cell-cycle regulation, inform neoplastic mechanisms, provide molecular biomarkers for refined diagnosis, and may suggest new opportunities for therapy. Whether other salivary gland neoplasms also harbor recurrent gene fusions has remained an open question.

Acinic cell carcinoma (AcCC) of the salivary gland is a malignant epithelial neoplasm named for its characteristic acinic cell differentiation [11]. AcCC accounts for approximately 5–11% of all salivary gland carcinomas and is considered as a low-grade malignancy, with a five-year disease-specific survival rate of 91% and a lifetime disease-associated death rate of 16% [12, 13]. Little is known about the pathogenesis of AcCC. Furthermore, ancillary tests and the immunoprofile of AcCC are non-specific and thus, diagnosis is made based primarily on morphologic criteria.

Here, to discover pathogenic gene fusions in AcCC, we performed whole transcriptome analysis of three prototypic cases. We report the identification of a novel gene fusion, *HTN3-MSANTD3*, and recurrent rearrangements of *MSANTD3* in 16% of AcCC. A hitherto unstudied gene, we also here surveyed MSANTD3 expression across a diverse set of human tissues and neoplasias, and carried out preliminary studies of its function.

## Materials and methods

### Tissue specimens

Salivary gland acinic cell carcinoma specimens were identified by an electronic search of the Stanford University Department of Pathology archives. In all, we retrieved formalin-fixed paraffin-embedded (FFPE) blocks for 27 cases dating between 1997 and 2014. Hematoxylin and eosin (H&E) stained tissue sections were reviewed by a pathologist (R.B.W) to confirm the diagnosis. Three AcCC cases were used for the RNA-seq studies, while all 27 were incorporated into a tissue microarray (TMA). All existing archived tissue specimens reported in this publication were used with Stanford University Institutional Review Board approval and HIPAA compliance (with waiver of informed consent based on minimal risk and impracticality).

### Transcriptome sequencing (RNA-seq)

RNA-seq of FFPE specimens was done as previously reported [14]. Briefly, FFPE blocks were sectioned (10μM thickness), RNA isolated using the Allprep RNA/DNA FFPE kit (Qiagen), and RNA quality verified by Agilent Bioanlyzer. Sequencing libraries (150 bp average insert size) were then prepared from 100 ng of rRNA-depleted RNA using TruSeq RNA Sample Preparation Kit v2 (Illumina), with four indexed libraries loaded per flow-cell lane. Paired-end 75-bp sequencing was carried out on a HiSeq 2000 instrument (Illumina), with the three AcCC libraries yielding 103–110 million sequencing reads. For discovery of gene fusions, evidenced by paired reads mapping to two different genes, and/or reads spanning predicted fusion junctions, we used Chimeriscan [15], TopHat-Fusion [16], SnowShoes-FTD [17], and deFuse [18] software. RNA-seq reads are available at GEO (accession GSE76354). Analysis of publically-available The Cancer Genome Atlas (TCGA) RNA-seq data was done using Wanderer [19], a web-based interactive viewer.

### Tissue microarrays

A TMA comprising 27 AcCC cases was constructed as previously described [20], using a manual tissue arrayer (Beecher Instruments). Each case was represented by two 0.6mm cores. Ten additional TMAs, covering other salivary gland tumor diagnoses as well as normal and neoplastic tissues from diverse anatomic sites, and together representing 1495 cases, were previously described [21, 22].

### Fluorescence *in situ* hybridization

Identified gene rearrangements were validated in TMA tissue sections by “break apart” FISH assay. Custom FISH probes for the *MSANTD3* locus were generated from bacterial artificial chromosomes (BAC) flanking *MSANTD3*, CTD-3186I20 Cy5 (telomeric) and CTD-2363K7 Cy3 (centromeric) (BACPAC Resources Center, Children’s Hospital Oakland Research Institute). Custom FISH probes for the *ZNF217* locus were CTD-2552P20 Cy5 (telomeric) and CTD-2511E9 Cy3 (centromeric). Break-apart FISH was done as previously described [22]. Briefly, TMA sections (6μm) were pretreated with citric acid buffer (pH 6.0). BACs were directly labeled with either Cy5 or Cy3 (GE Healthcare Life Sciences) and then hybridized using Vysis reagents and protocols. Slides were counterstained with 4,6-diamidino 2-phenylindole for microscopy, and images captured using Ariol software (Applied Imaging). For each case, 100 nuclei were evaluated and cases scored positive for rearrangement if ≥30 breaks of the two probes were present. Cases with poor FISH probe hybridization were considered unevaluable, and excluded from subsequent analysis.

### Immunohistochemistry

MSANTD3 protein expression was evaluated by immunohistochemistry using an anti-MSANTD3 antibody (LS-C146308, 1:2400 dilution; LifeSpan Biosciences) and peroxidase-based chromogenic staining (EnVision, Dako). All slides were uploaded to the Stanford Tissue Microarray Database [23]. Cores were evaluated for nuclear staining and assigned a score of strong (2), moderate (1), weak (+/-), negative (-), or uninterpretable/missing core (<100 evaluable nuclei). Strong staining was defined as intense staining in >30% or faint staining in > 70% of nuclei; moderate staining as intense staining in 5–30% or faint staining in 30–70% of nuclei; weak staining as intense staining in 0–5% or faint staining in 10–30% of nuclei; and negative staining as faint staining in <10% of nuclei. Images were independently evaluated using color segmentation software (GemIdent). Briefly, GemIdent was trained to recognize positive and negative nuclei, and a percentage of positive staining was calculated. Since GemIdent could not readily distinguish intensities, the algorithm of < 9% staining was defined as negative, 9–14% as weak, 14–26% as moderate, and >26% as strong. Discrepancies between the manual and automated scores were further reviewed and a final score assigned by the pathologist.

### Phylogenetic studies

Conserved protein domains within MSANTD3 were identified by search of NCBI’s conserved domain database [24]. Local protein sequence alignments were performed using the Multiple Alignment Construction and Analysis Workbench (MACAW) [25]. Global protein sequence-alignments were performed using ClustalX [26]. A boot-strapped phylogenetic tree was displayed with TreeView [27].

### Cell culture studies

SMG-C6 cells, an SV40-immortalized rat submandibular salivary gland acinar cell line [28] (kind gift of Dr. Margarita M. Vasquez, University of Texas Health Science Center at San Antonio), were grown in DMEM/F12 media supplemented as described [29]. NIH-3T3 mouse embryo fibroblasts were obtained from the ATCC and grown in DMEM media supplemented with 10% bovine calf serum. A human C-terminally Myc-tagged MSANTD3 cDNA ORF clone was obtained from Origene (SKU:RC203850), and then subcloned into pLentiCMV-Puro (Addgene #17452) to create pLentiCMV-MSANTD3. pLentiCMV-MSANTD3 or empty vector control was packaged in 293T cells (ATCC) using ViraPower Lentiviral Packaging Mix (Thermo Fisher Scientific), and then transduced into SMG-C6 cells and selected with 4 μg/ml puromycin. Western blotting was done as previously described [30], using anti-MSANTD3 antibody (LS-C146308, 1:1000 dilution; LifeSpan Biosciences) and anti-Myc antibody (9B11, 1:500 dilution; Cell Signaling). For cell proliferation assays, cells were plated in 6-well plate wells (20,000 cells per well in triplicate) and cell proliferation determined 1, 3 and 5 days later by Wst-1 assay (Roche). For contact-inhibition studies, NIH-3T3 cells were transduced with pLentiCMV-MSANTD3, KRAS(V12) positive-control, or empty vector control as above, and selected in 2 μg/ml puromycin. One million cells were plated per 10 cm dish in triplicate, and 16 days later cells were fixed with methanol, stained with 0.5% crystal violet (in 25% methanol), and then foci ≥3 mm counted. For SMG-C6 transcriptome studies, RNA was isolated with RNeasy (Qiagen), and then the TruSeq RNA Sample Preparation Kit v2 (Illumina) used to prepare barcoded-sequence libraries, which were sequenced on an Illumina HiSeq2000 instrument (101bp paired reads) to 36–49 million reads. Reads were aligned to the rat genome using DNAnexus, which also provided reads per kilobase transcript per million mapped reads (RPKM). Non-expressed genes (RPKM<1) were filtered, and gene RPKM ratios (MSANTD3/empty vector) calculated. Differentially-expressed gene sets were then identified using Gene Set Enrichment Analysis (GSEA) [31], with the ‘pre-ranked’ option and default settings. RNA-seq reads are available at GEO (accession GSE76354).

## Results

### Transcriptome sequencing identifies novel gene fusions in acinic cell carcinoma

As part of a broader transcriptome survey to discover novel oncogenic gene fusions or mutations in less common cancer diagnoses that are available mainly as archived FFPE tissue blocks [14], here we carried out whole-transcriptome sequencing of three prototypic AcCC cases. Illumina sequencing libraries were constructed from rRNA-depleted total RNA, and Illumina paired-end sequencing (75bp x 2) was done to a depth of 103–110 million reads per case. To discover gene fusions, evidenced by paired sequencing reads mapping to two different genes and/or reads spanning predicted fusion junctions, we used four different software tools: Chimeriscan [15], TopHat-Fusion [16], SnowShoes-FTD [17], and deFuse [18]. All four algorithms identified a total of two fusion genes, both novel, and one in each of two different AcCC cases.

**Fig 1:**
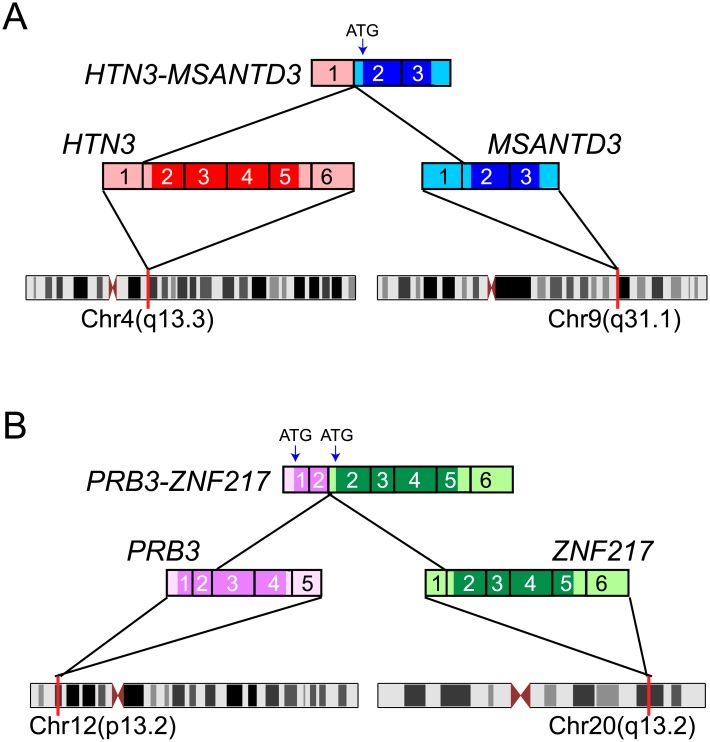
Novel genes fusions in acinic cell carcinoma. **(A)** Predicted structure of the *HTN3*-*MSANTD3* fusion gene. Exon 1 (non-coding) of *HTN3* is fused to the exon 2 (first coding exon) of *MSANTD3*, leading to predicted overexpression of full-length MSANTD3 protein (translation start site is indicated). Fusion junction-spanning sequence reads are shown in S1 Fig. **(B)** Predicted structure of the *PRB3*-*ZNF217* gene fusion. Here, exon 2 (coding) of *PRB3* is fused to exon 2 (first coding) of *ZNF217*, possibly leading to the overexpression (by internal initiation of translation) of full-length ZNF217 protein. Fusion junction-spanning sequence reads are shown in S2 Fig.

The first novel chimeric transcript resulted from fusion of noncoding exon 1of *Histatin 3* (*HTN3*; cytoband 4q13.3) to exon 2 (the first coding exon) of *Myb/SANT-like DNA-binding domain contain 3* (*MSANTD3*; cytoband 9q31.1) (Fig 1A). The fusion junction was supported by 51 junction-spanning reads (with 28 unique reads; S1 Fig). Consequent to the rearrangement, the promoter of a highly-expressed salivary gland gene, the microbial peptide precursor *HTN3* [32], ostensibly drives overexpression of MSANTD3, an uncharacterized protein harboring a Myb/SANT-like domain characteristic of the MYB oncoprotein and SANT family proteins [33].

The second chimera resulted from fusion of exon 2 of *Proline-rich protein BstNI subfamily 3* (*PRB3*; cytoband 12p13.2) to exon 2 (the first coding exon) of *Zinc finger protein 217* (*ZNF217*; cytoband 20q13.2) (Fig 1B). The fusion junction was supported by 78 junction-spanning reads (with 36 unique reads; S2 Fig). The rearrangement results in the promoter of another highly-expressed salivary gland gene, *PRB3* [34], possibly driving overexpression of ZNF217, a reported oncoprotein in several cancer types [35]. However, the start codon (ATG) of *ZNF217* is out of frame relative to the normal *PRB3* open reading frame (Fig 1B), and therefore generation of ZNF217 protein would necessitate internal initiation of translation.

### FISH analysis reveals recurrent *MSANTD3* rearrangement in acinic cell carcinoma

**Fig 2:**
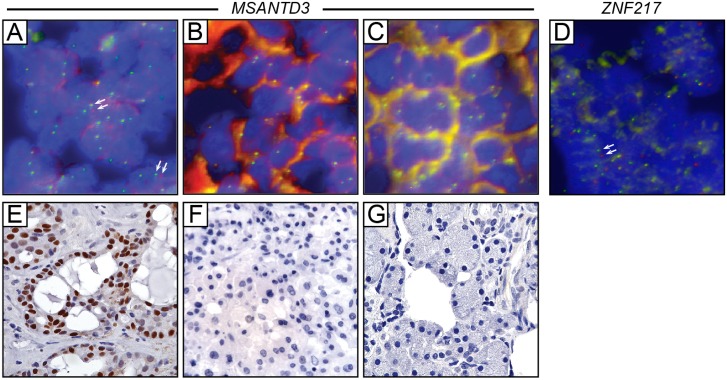
Evaluation of MSANTD3 rearrangement and protein expression in accinic cell carcinoma tissue sections. **(A-C)** Break-apart FISH assay of *MSANTD3* locus rearrangement; green FISH probe is flanking centromeric and red FISH probe is flanking telomeric to *MSANTD3*. Note positive rearrangement (evidenced by physically-separated green and red signals, arrows) in AcCC case **(A)**. AcCC case **(B)** is negative for the rearrangement (i.e., signals co-localize), as is observed in normal salivary gland control **(C)**. **(D)** Break-apart FISH assay of *ZNF217* rearrangement, confirming the rearrangement in the index case. **(E-G)** Corresponding MSANTD3 immunostaining of the above cases. Note the strong nuclear MSANTD3 staining **(E)** corresponding to the FISH-positive rearranged specimen.

To validate *MSANTD3* rearrangement in the index case, and to identify possible *MSANTD3* rearrangements in additional AcCC cases, we developed a ‘break-apart’ FISH assay. In this assay, differentially fluorescently-labeled FISH probes flanking the *MSANTD3* locus would appear co-localized in normal interphase nuclei, but physically-separated in nuclei harboring allelic rearrangement at the locus. We carried out break-apart FISH on a tissue microarray comprising 27 different AcCC cases, including the three cases evaluated by RNA-seq. In all, 3 of the 20 evaluable cases (including the index case) exhibited break-apart rearrangement of *MSANTD3* locus (Table 1 and Fig 2A–2C), validating the rearrangement in the index case, and estimating the recurrence rate to be 15%. In all three FISH-positive cases, the rearrangement was heterozygous (i.e. affecting only one *MSANTD3* allele), consistent with a dominantly acting oncogene. A fourth AcCC case showed isolated loss of the telomeric FISH probe (downstream of *MSANTD3*) (Table 1), a finding with uncertain expected impact on MSANTD3 expression.

**Table 1:** Acinic cell carcinoma cases.

Case^a^	*MSANTD3* FISH	MSANTD3 IHC score	*ZNF217* FISH	RNA-seq fusion	Lymphocytic infiltrate^b^
1	BREAK APART	Strong	Normal	*HTN3*-*MSANTD3*	No
2	BREAK APART	Strong			No
3	BREAK APART	Strong			No
4	Normal	Strong			YES
5	Normal	Strong			YES
6	Normal	Strong			No
7		Strong			No
8	Not scorable	Strong	BREAK APART	*PRB3*-*ZNF217*	No
9	Normal	Moderate	Normal		YES
10	Normal	Moderate			YES
11	Normal	Moderate	Normal		No
12	Not scorable	Moderate	Normal		No
13	Normal	Weak	Normal		YES
14	Normal	Weak	Normal		YES
15	Normal	Weak	Normal		YES
16	Normal	Weak	Normal		No
17	Normal	Weak			No
18	Not scorable	Weak	Normal		No
19	Loss of red signal	Negative	Normal		No
20	Normal	Negative	Normal	None identified	No
21	Normal	Negative	Normal		No
22	Normal	Negative			No
23	Normal	Negative			No
24	Normal	Negative			No
25	Not scorable	Negative	Normal		No
26		Negative			No
27		Negative			No

^a^Ordered by MSANTD3 IHC score

^b^Defined as lymphocytic infiltrate greater than 30%

We developed an analogous ‘break-apart’ FISH assay for the *ZNF217* locus. In all, 13 of the 27 cases were evaluable (i.e. sufficient FISH hybridization signal), and only the index case scored positive for *ZNF217* (heterozygous) locus rearrangement (Fig 2D). Thus, while we validated rearrangement in the index case, we did not find evidence for recurrent rearrangement. As such, our subsequent studies focused on the recurrent *MSANTD3* rearrangement.

### MSANTD3 is highly expressed in a subset of acinic cell carcinomas

In the index case, the *HTN3* promoter ostensibly drives the overexpression of a chimeric transcript encoding full-length MSANTD3 protein. To evaluate MSANTD3 protein expression, we carried out immunohistochemistry on the above TMA. Of 27 AcCC cases, 8 (30%) were strongly positive for MSANTD3 diffuse nuclear-staining (the expected cellular compartment for Myb/SANT domain-containing proteins) (Table 1 and Fig 2E–2G). The 8 IHC strongly-positive cases included all 3 cases with *MSANTD3* rearrangement, a statistically-meaningful enrichment (*P* = 0.02; two-tailed Fisher’s exact test). Also noteworthy, 7 of the 27 AcCC cases demonstrated extensive lymphocytic infiltration (a common finding in AcCC; [36]), and all 7 were among the 18 cases that expressed MSANTD3 to some degree (Table 1) (*P* = 0.06, two-tailed Fisher’s exact test); albeit, all 7 cases were FISH-negative for *MSANTD3* rearrangement. The meaning of this association is unclear.

### MSANTD3 expression varies across other neoplasias of the salivary gland and diverse tissue types

Since MSANTD3 was an unstudied protein, we sought to characterize its expression across a wider array of benign and malignant tissues. Towards this goal, we carried out IHC staining of 11 previously described TMAs, altogether comprising 1293 evaluable cases representing normal and neoplastic tissues of the salivary gland, brain, head and neck, gastrointestinal, cardiovascular, respiratory, immunologic, endocrine, integumentary and musculoskeletal systems. Of these, 258 cases were of salivary origin, and the remaining 1035 cases were from other organ systems and included 235 normal tissues, 70 embryonic tissues, 55 benign or pre-malignant tissues, 40 benign neoplasms and 635 malignant neoplasms.

**Fig 3:**
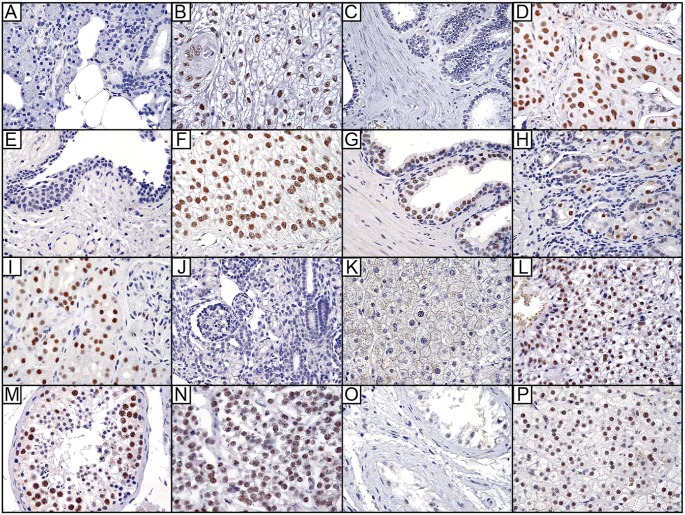
Representative images of MSANTD3 immunostaining in select tissues. **(A)** Normal salivary tissue, **(B)** Mucoepidermoid carcinoma, **(C)** Normal breast tissue, **(D)** Invasive breast carcinoma, not otherwise specified, **(E)** Normal bladder, **(F)** Urothelial carcinoma, **(G)** Normal prostate, **(H)** Normal stomach, **(I)** Normal kidney, **(J)** Fetal kidney, **(K)** Normal liver, **(L)** Hepatocellular carcinoma, **(M)** Normal testicle, **(N)** Seminoma, **(O)** Testicular atrophy, and **(P)** Adrenal cortex. Images were acquired at 40x magnification.

With respect to the salivary gland tissues (Table 2 and Fig 3A and 3B), MSANTD3 was expressed in normal salivary gland ductal cells to varying degrees. However, acinar cells were most often entirely negative (24/33 cases), or displayed weak staining (5/33) and at most moderate staining (4/33). While 51% of salivary specimens (benign and malignant) tested were void of any MSANTD3 staining, strong MSANTD3 expression was observed in a substantial subset of mucoepidermoid carcinomas (5/25, 20%) and adenosquamous carcinomas (3/5, 60%). FISH performed on 120 of the salivary gland neoplastic cases (other than AcCC) identified no additional *MSANTD3* rearrangements. To exclude the possibility that our MSANTD3 antibody might cross react with MYB (itself rearranged in a subset of salivary gland tumors), IHC-staining for MYB was performed and compared with MSANTD3 staining across 172 cases and no correlation was observed (data not shown).

**Table 2:** MSANTD3 immunostaining of salivary gland benign and malignant specimens.

		Immunostaining score (number of cases, %)								
Salivary gland		Strong	%	Moderate	%	Weak	%	Negative	%	Total
Normal	Adult	0	0%	4	12%	5	15%	24	73%	33
	Embryonic	0	0%	0	0%	0	0%	2	100%	2
Benign	Pleomorphic Adenoma	2	4%	5	10%	11	22%	32	64%	50
	Basal Cell Adenoma	0	0%	0	0%	3	33%	6	67%	9
	Monomorphic Adenoma	0	0%	0	0%	0	0%	1	100%	1
	Oncocytoma	1	9%	2	18%	4	36%	4	36%	11
	Oncocystic Hyperplasia	2	67%	0	0%	1	33%	0	0%	3
	Oncocytic Cystadenoma	0	0%	0	0%	1	100%	0	0%	1
	Myoepithelioma	0	0%	0	0%	1	17%	5	83%	6
	Warthin Tumor	1	100%	0	0%	0	0%	0	0%	1
Malignant	*AcCC*: *MSANTD3 Rearrangement*	3	100%	0	0%	0	0%	0	0%	3
	*AcCC*: *Normal MSANTD3 FISH*	3	18%	3	18%	5	29%	6	35%	17
	*AcCC*: *MSANTD3 FISH* not evaluable	2	29%	1	14%	1	14%	3	43%	7
	Adenocarcinoma NOS	1	8%	1	8%	3	23%	8	62%	13
	Adenoid Cystic Carcinoma	3	5%	13	22%	15	25%	28	47%	59
	Adenosquamous Carcinoma	3	60%	0	0%	1	20%	1	20%	5
	Basal Cell Adenocarcinoma	0	0%	0	0%	0	0%	1	100%	1
	Mucoepidermoid	5	20%	8	32%	5	20%	7	28%	25
	Myoepithelial	0	0%	1	20%	1	20%	3	60%	5
	Salivary Duct Carcinoma	1	33%	2	67%	0	0%	0	0%	3
	NOS	0	0%	1	33%	1	33%	1	33%	3
	**Total**	27	10%	41	16%	58	22%	132	51%	258

Beyond the salivary gland (Table 3 and Fig 3C–3P), MSANTD3 was expressed in a small subset of normal cell types and in many neoplasms. In normal tissues, expression was identified in spermatogonia and early spermatocytes, the parietal cells of stomach, tubular and parietal epithelial cells of the kidney, cortical cells of the adrenal gland, uterine endometrium and the glandular epithelium of the prostrate.

**Table 3:** MSANTD3 immunostaining across diverse benign and neoplastic tissues.

	Tissue	MSANTD3 strong immunostaining (No. cases)	Total No. cases	% strongly positive
Testis	*Normal*	14	14	100%
	Atrophic/Immature	0	4	0%
	Seminoma	19	22	86%
	Teratoma	2	3	67%
	Yolk Sac Tumor	4	4	100%
	Embryonal Carcinoma	1	2	50%
	Granulosa Cell Tumor	1	1	100%
	Leydig Cell Tumor	0	1	0%
Adrenal	*Normal Cortex*	14	22	64%
	Adenoma	1	3	33%
	Adrenocortical Carcinoma	1	2	50%
	Pheochromocytoma	0	2	0%
Bladder	*Normal*	0	7	0%
	Embryonic	0	2	0%
	Adenocarcinoma	2	3	67%
	Uroethelial Carcinoma	3	14	21%
Breast	*Normal*	0	7	0%
	Invasive Ductal	4	11	36%
	Invasive Lobular	2	3	67%
	Invasive NOS	75	248	30%
Kidney	*Normal*	11	13	85%
	Embryo	0	5	0%
	Oncocytoma	0	2	0%
	Clear Cell Carcinoma	0	1	0%
	Conventional Carcinoma	0	4	0%
	Papillary Carcinoma	0	4	0%
Liver	*Normal*	0	11	0%
	Hepatocellular	4	10	40%
Lung	Normal	0	7	0%
	Andenocarcinoma	8	14	57%
Ovary	Normal	0	7	0%
	Mucinous Carcinoma	2	3	67%
	Clear Cell Carcinoma	3	4	75%
	Serous Carcinoma	3	5	60%
Prostate	*Normal*	3	6	50%
	Adenocarcinoma	7	12	58%
Stomach	*Normal*	3	10	30%
	Adenocarcinoma	5	7	71%
Thyroid	*Normal*	0	12	0%
	Papillary Carcinoma	2	5	40%
	Follicular Carcinoma	1	2	50%
Pancreas	*Normal*	0	3	0%
	Adenocarcinoma	1	2	50%
	Ductal	2	3	67%
Uterus	Endometrium	2	3	67%
	Endometrial Carcinoma	7	11	64%
	Leiomyosarcoma	0	5	0%

Expression in carcinoma was more prominent, with strong expression in multiple gonadally-derived carcinomas, adenocarcinomas of the stomach, lung, pancreas, endometrium, and bladder, as well as, papillary thyroid carcinoma, hepatocellular carcinoma, urothelial carcinoma and a subset of invasive breast cancers. In contrast, MSANTD3 expression was rarely observed in normal and neoplastic tissues of the brain, head and neck region, esophagus, integument, musculoskeletal system, lymphovascular system, small bowel and large bowel. Also, tubularly-derived carcinomas of the kidney did not exhibit staining, an interesting finding considering the prominent expression in normal kidney tubules. Analysis of publicly-available The Cancer Genome Atlas (TCGA) RNA-seq data also showed varied MSANTD3 transcript levels across cancer types, with several exhibiting significantly higher expression in cancer compared to matched normal tissue (S3 Fig).

In addition, because we had stained a large number of breast cancers and had clinical outcome parameters on these specimens, we attempted to identify clinicopathologic correlates. We found no correlation between MSANTD3 expression and ER or HER2 status, nor did we identify a significant correlation with survival (*P* = 0.18, log-rank test).

### MSANTD3 protein is highly conserved among tetrapods

**Fig 4:**
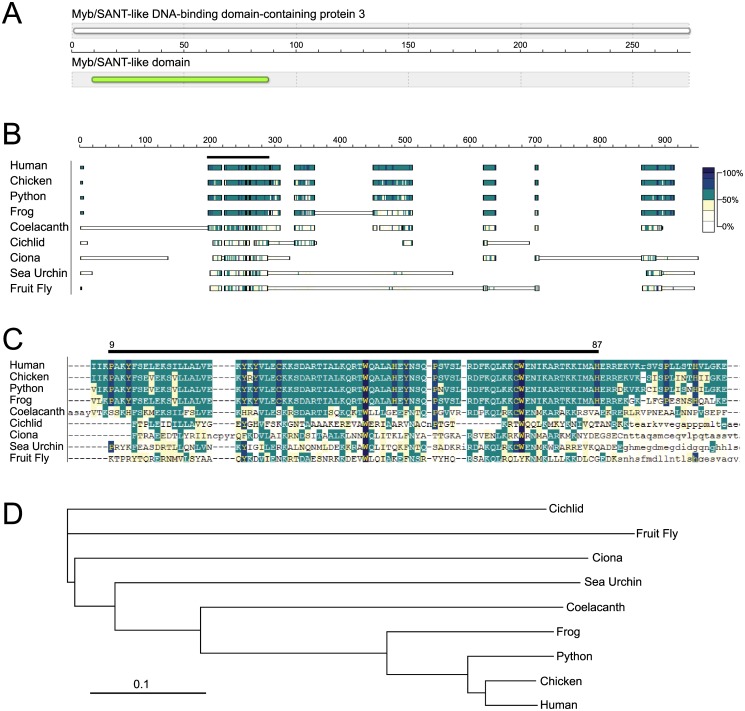
MSANTD3 structural domains and phylogenetic conservation. **(A)** Schematic depiction of MSANTD3 domains, showing the location of the Myb/SANT-like domain within the N-terminus. **(B)** Schematic depiction of MSANTD3 MACAW sequence alignments across species. Boxes indicate conserved blocks, while the shading indicates pair-wise scores relative to human MSANTD3 with colors indicated in the key. *Above*, the horizontal black bar indicates the location of the conserved Myb/MSANT domain found by NCBI search (Pfam 13873). **(C)** Actual MACAW alignment within the MYB/SANT region. **(D)** Phylogenetic tree based on the global alignment made by ClustalX and visualized using Treeview software.

Because little was known about MSANTD3, we also examined its evolutionary conservation to infer function and to identify possible relevant model organisms. Human MSANTD3 comprises 275 amino acids, with a predicted molecular weight of 32 kD. A search for conserved protein domains identified only the Myb/SANT domain within the N-terminus (Fig 4A). Analysis of amino acid conservation across species revealed MSANTD3 to be highly conserved in mammals (human), birds (chicken), reptiles (python), and amphibians (frog), but far less conserved in ray-finned fish (cichlid) (Fig 4B–4D). Even more distantly related MSANTD3-like proteins are present in invertebrates (*Ciona*, sea urchin, and fruit fly). There appeared to be no *MSANTD3*-related gene in nematode worms, plants, fungi, or bacteria. The *MSANTD3* homolog in cichlids (bony fish) is about as distant from other vertebrates as is the insect gene. Other fish (e.g. fugu and zebrafish) have even more distantly related genes. Interestingly, the coelacanth, a lobe-finned fish that is thought to be related to a common ancestor of all tetrapods [37], has an MSANT3 protein more similar to those of land vertebrates. Furthermore, the vertebrate and coelacanth proteins share homologous regions outside of the N-terminal Myb/SANT domain that are poorly conserved in other species.

### MSANTD3 overexpression upregulates genes functioning in protein synthesis

**Fig 5:**
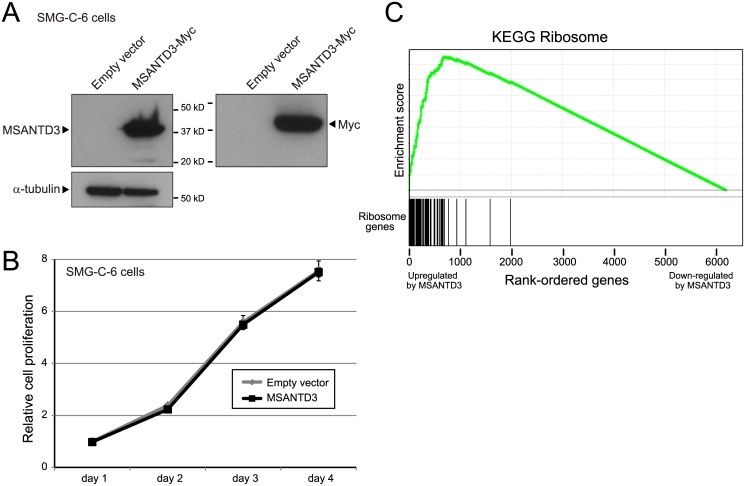
MSANTD3 overexpression leads to the upregulation of genes involved in protein synthesis. **(A)** Overexpression of C-terminally Myc-tagged MSANTD3 in SMG-C6 immortalized rat salivary gland epithelial cells, demonstrated by western blot using anti-MSANTD3 antibody (*left*) and anti-Myc tag antibody (*right*). Note, the observed band runs close to the calculated MW of the tagged protein (36 kD). **(B)** MSANTD3 overexpression does not enhance cell proliferation, compared to empty vector control, quantified by Wst-1 assay. **(C)** Transcriptome (RNA-seq) analysis of MSANTD3 overexpression identifies significant upregulation of gene sets associated with protein synthesis. Gene Set Enrichment Analysis (GSEA) of the top gene set (‘KEGG Ribosome’) is shown; other significant gene sets are listed in S1 Table. Note, the early and positive upswing of the Enrichment profile reflects the early concentration of ribosome genes within the ranked list of genes upregulated by MSANTD3 overexpression (compared to empty vector control).

Recurrent rearrangement of *MSANTD3* in AcCC, ostensibly driving overexpression of the full-length MSANTD3 protein, combined with observed overexpression by IHC, strongly suggested an oncogenic driver role in AcCC. To preliminarily characterize potential oncogenic functions, we used lentiviral transduction to engineer MSANTD3 overexpression in cultured cells, first using an immortalized rat submandibular salivary gland acinar cell line model [28], SMG-C6. Overexpression of MSANTD3 in SMG-C6 cells, confirmed by western blot (Fig 5A) did not enhance cell proliferation (Fig 5B). Nor did overexpression of MSANTD3 in NIH-3T3 mouse embryo fibroblast cells result in lost contact-inhibition (assayed by focus formation; S4 Fig). To explore potential oncogenic phenotypes more broadly, we profiled transcriptomic changes by RNA-seq in SMG-C6 cells overexpressing MSANTD3, compared to empty vector control. By Gene Set Enrichment Analysis, MSANTD3 overexpression led to marked upregulation of multiple gene sets associated with protein synthesis, including ‘ribosome’, ‘peptide chain elongation’, and ‘translation’ (Fig 5C and S1 Table).

## Discussion

Recurrent chromosomal rearrangements (and the resultant gene fusions) are known to characterize other salivary gland tumors, but have not previously been reported in AcCC. Here, by whole-transcriptome profiling of three AcCC cases, we discovered two novel fusion genes, *HTN*-*MSANTD3* and *PRB3*-*ZNF217*, and found that *MSANTD3* locus rearrangements are recurrent events observed in approximately 15% of AcCC cases. Furthermore, in a cell line model, MSANTD3 overexpression led to the upregulation of genes involved in protein synthesis, a cellular process often upregulated in cancer [38].

Notably, both novel fusion genes juxtapose a highly-expressed salivary gland gene (*HTN3* or *PRB3*) to the full-length 3’ partner (*MSANTD3* and *ZNF217*, respectfully), ostensibly driving the overexpression of a chimeric transcript encoding the full-length 3’ fusion partner. For the *MSANTD3* rearrangement, we showed by immunostaining that MSANTD3 protein was in fact highly expressed in all three cases with DNA rearrangement. Though we note that for the other two cases with *MSANTD3* rearrangement, the FFPE tissue blocks were exhausted and so we did not have the opportunity to identify a putative 5’ fusion partner, which may or may not also be *HTN3*. Notably, MSANTD3 protein was highly expressed in an additional 5 cases (for 30% of all cases), presumably upregulated by some mechanism other than genomic DNA rearrangement.

With respect to the *PRB3*-*ZNF217* fusion, ZNF217 is a zinc finger protein, a protein family often functioning in transcriptional regulation through sequence-specific DNA binding [39]. ZNF217 was first characterized as the ‘driver’ oncogene amplified at 20q13.2 in breast cancer [40], where it was found to promote cellular immortalization [40]. Since then, ZNF217 has been implicated in ovarian and other cancer types, where it has been linked to diverse oncogenic phenotypes including cell proliferation, survival, and invasion [35]. Although we have not found *ZNF217* rearrangements in other AcCC cases, and we have not demonstrated ZNF217 protein expression, given its known oncogenic functions in other cancers it is intriguing to speculate a possible pathogenic role in a subset of AcCC cases.

In contrast, the recurrent nature of *MSANTD3* gene rearrangements provides strong genetic support for its pathogenic role. *MSANTD3* is a previously unstudied gene, named for harboring a protein domain with shared homology to the DNA-binding domains of MYB and SANT-family proteins. *MYB*, the homolog of the avian myeloblastosis viral oncogene, functions in normal hematopoiesis, and deregulated MYB expression has been linked with human leukemia [41]. SANT is an acronym for switching-defective protein 3 (**S**wi3), adaptor 2 (**A**da2), nuclear receptor co-repressor (**N**-CoR), transcription factor (**T**F)IIIB, with the four proteins (and others since) found to share a conserved 50 amino-acid motif with the MYB DNA-binding domain [33]. SANT domains are often present within subunits of chromatin-remodeling and histone-modifying complexes [42]. It was initially hypothesized that all SANT domains bind directly to DNA to enable their regulatory function. Structural and biochemical evidence for this model has been provided in at least some cases [43–45]. However, it has also been hypothesized that some SANT domains may bind directly to histones rather than to DNA in order to regulate chromatin structure and function [42].

Interestingly, in a recent yeast two-hybrid screen to identify binding partners of methyltransferase enzymes [46], MSANTD3 (formerly known as C9orf30) was identified as one of many putative binding partners of SUV39H1, itself a histone methyltransferase functioning in establishing heterochromatin [47]. This finding implicates a possible role of MSANTD3 in chromatin regulation, a function consistent with its having a Myb/SANT-like domain. We also note that the observed immunostaining was predominantly nuclear, also consistent with a role in chromatin dynamics. Nonetheless, detailed mechanistic studies will be necessary to elucidate the protein’s normal function.

To further explore MSANTD3 biology, we carried out a broad immunostaining survey of diverse normal and neoplastic tissues. Within the normal salivary gland, MSANTD3 generally showed higher expression in ductal compared to acinar cells. MSANTD3 was also expressed in a subset of other salivary gland neoplasias, though without evidence of locus rearrangement. Other tissues that stained strongly included normal testis and kidney, and glandular tissues including prostate and endometrium. Adenocarcimomas of diverse organ sites showed varied staining, while squamous cell carcinomas, sarcomas, and lymphoid-derived neoplasias were generally void of staining. The expression of MSANTD3 across diverse tissue types, as well as its protein conservation down to amphibians, suggests an important cellular function(s). Those functions, as well as possible roles in diseases beyond AcCC, remain to be explored.

To further characterize its pathogenic role in AcCC, we overexpressed MSANTD3 in SMG-C6 immortalized rat salivary gland epithelial cells, a reasonable approximation of a normal cell type equivalent. MSANTD3 overexpression did not enhance cell proliferation. However, by expression profiling we found that its overexpression led, either directly or indirectly, to marked upregulation of genes involved in translation of mRNA into proteins by ribosomes. This finding is intriguing because increased protein synthesis is a common feature of cancers [38]. Oncoproteins including PI3-kinases, AKT kinase, ribosomal S6 kinase, and MYC are all thought to act in large part by enhancing ribosome biogenesis and translation, to provide needed new proteins for cell growth and proliferation [48, 49].

Of note, in a recent RNA-seq survey of cancer cell lines, a *XRCC4*-*MSANTD3* in-frame fusion was reported in SW780 bladder transitional cell carcinoma cells [50] (their Supplementary Table 8). However, that fusion was supported by only two sequencing reads, and the predicted fusion incorporates only the terminal exon of *MSANTD3* and so would be missing the highly-conserved Myb/SANT-like domain), and thus is unlikely to be functional.

Although the exact role(s) of MSANTD3 in AcCC oncogenesis remain to be determined, it is also intriguing that MYB itself, which of course also harbors a Myb/SANT-like domain, is part of a gene fusion that characterizes a different salivary gland tumor: *MYB*-*NFIB* in adenoid cystic carcinoma. Perhaps this finding suggests a more general role of Myb/SANT-like DNA-binding domain containing proteins in the normal biology and neoplastic conversion of salivary epithelium.

From a diagnostic standpoint, several salivary gland neoplasias have been found to harbor specific gene fusions that have proven to be useful biomarkers for differential diagnosis [6]. In our study, we have identified *MSANTD3* rearrangements in 15% of AcCC specimens, and in no other salivary gland neoplasias. While its modest frequency in AcCC would limit its overall diagnostic utility, finding *MSANTD3* rearrangement (e.g. by FISH) in a diagnostically challenging case could nonetheless provide support for a diagnosis of AcCC. Whether *MSANTD3* rearrangement and/or overexpression confer a distinct prognosis remains unanswered, and need await studies on larger cohorts with clinical follow up.

In summary, by whole-transcriptome sequencing of archival AcCC cases and validation by FISH, we have discovered novel gene fusions including a recurrent rearrangement of the previously uncharacterized gene *MSANTD3*. Functional studies implicate a role of MSANTD3 in upregulating translation; however, the normal and tumorigenic mechanisms of this novel putative oncogene await future investigation. Based on our studies, AcCC now joins other salivary gland neoplasias in harboring specific chromosome rearrangements, and may ultimately aid in the diagnosis and understanding of disease pathogenesis.

## Supporting information

S1 Fig *HTN3*-*MSANTD3* junction-spanning reads. Shown are the 51 sequencing reads spanning the predicted *HTN3*-*MSANTD3* junction, identified from the Chimeriscan analysis.
(PDF)

S2 Fig *PRB3*-*ZNF217* junction-spanning reads. Shown are the 78 sequencing reads spanning the predicted *PRB3*-*ZNF217* junction, identified from the Chimeriscan analysis.
(PDF)

S3 Fig MSANTD3 transcript levels vary across cancer types. Plots display normalized transcript levels for cancer *vs*. normal samples, shown for those TCGA cancer types where there are sufficient sample numbers to perform a statistical analysis (tumor *vs*. normal; Wilcoxon *P*-value indicated). Plots assembled using Wanderer (see Methods).
(PDF)

S4 Fig MSANTD3 overexpression does not lead to loss of contact-inhibition (focus formation). **(A)** Focus formation assay in NIH-3T3 cells transduced with pLenti-CMV-MSANTD3 (*vs*. pLenti-CMV empty vector control), or positive control pBABE-KRAS(V12) (*vs*. pBABE empty vector control). Crystal violet stained foci were manually counted in triplicate 10cm plates; representative plates shown. **(B)** Graphical display of counted foci. Note, no foci (>3mm) were observed following MSANTD3 overexpression.
(PDF)

S1 Table Canonical pathway gene sets found enriched by GSEA analysis. (PDF)
