# Supplementary material for: Recurrent rearrangements of the Myb/SANT-like DNA-binding domain containing 3 gene (MSANTD3) in salivary gland acinic cell carcinoma
Source: PLoS One. 2017 Feb 17;12(2):e0171265. doi: 10.1371/journal.pone.0171265 (PMC5315303; doi:10.1371/journal.pone.0171265)
Supplement: S1 Fig — Shown are the 51 sequencing reads spanning the predicted HTN3-MSANTD3 junction, identified from the Chimeriscan analysis. (PDF) [file pone.0171265.s001.pdf]

S1 Fig. *HTN3*-*MSANTD3* junction-spanning reads

```
  ←---------------------------HTN3 exon 1-----------------------------||-----------------------MSANTD3 exon 2--------------------------→
5'-TAGGAAAGGACATCTCTTGAGACTTCACTTCAGCTTCACTGACTTCTGGATTCTCCTCTTGAGTAAAAGGATAGCTAGCGGCCAGGAGAAATACAGTGGAAAATGCAAAACAACGAAATTATAAAGCCTGCCAAA-3'

Fusion junction-spanning reads:
   TAGGAAAGGACATCTCTTGAGACTTCACTTCAGCTTCACTGACTTCTGGATTCTCCTCTTGAGTAAAAGGATAGC
   TAGGAAAGGACATCTCTTGAGACTTCACTTCATCTTCACTGACTTCTGGATTCTCCTCTTGAGTAAAAGGATAGC
     GGAAAGGACATCTCTTGAGACTTCACTTCAGCTTCACTGACTTCTGGATTCTCCTCTTGAGTAAAAGGATAGCTA
      GAAAGGACATCTCTTGAGACTTGACTTCAGCTTCACTGACTTCTGGATTCTCCTCTTGAGTAAAAGGATAGCTAG
          GGACATCTCTTGAGACTTCACTNCAGCTTCACTGACTTCTGGATTCTCCTCTTGAGTAAAAGGATAGCTAGCGGC
          GGACATCTCTTGAGACTTCACTTCAGCTTCACTGACTTCTGGATTCTCCTCTTGAGTAAAAGGATAGCTAGCGGC
                  CTTGAGACTTCACTTCAGCTTCACTGACTTCTGGATTCTCCTCTTGAGTAAAAGGATAGCTAGCGGCTAGGAGAA
                  CTTGAGACTTCACTTCAGCTTCACTGACTTCTGGATTCTCCTCTTGAGTAAAAGGATAGCTAGCGGCCAGGAGAA
                   TTGAGATTTCACTTCAGCTTCACTGACTTCTGGATTCTCCTCTTGAGTAAAAGGATAGCTAGCGGCCAGGAGAAA
                   TTGAGACTTCACTTCAGCTTCACTGACTTCTGGATTCTCCTCTTGAGTAAAAGGATAGCTAGCGGCCAGGAGAAA
                    TGAGACTTCACTTCAGCTTCACTGACTTCTGGATTCTCCTCTTGAGTAAAAGGATAGCTAGCGGCCAGGAGAAAT
                         CTTCACTTCAGCTTCACTGACTTCTGGATTCTCCTCTTGAGTAAAAGGATAGCTAGCGGCCAGGAGAAATACAGT
                         NTTCACTTCAGCTTCACTGACTTCTGGATTCTCCTCTTGAGTAAAAGGATAGCTAGCGGCCAGGAGAAATACAGT
                         CTTCACTGCAGCTTCACTGACTACTGGATTCTCCTCTTGAGTAAAAGGATAGCTAGCGGCCAGGAGAAATACAGT
                         CTTCACTTCAGCTTCACTGACTTCTGGATTCTCCTCTTGAGTAAAAGGATAGCTAGCGGCCAGGAGAAATACAGG
                         CTTCACTTCAGCTTCACTGACTNCTGGATTCTCCTCTTGAGTAAAAGGATAGCTAGCGGCCAGGAGAAATACAGT
                          TTCACTTCAGCTTCACTGACTTCTGGATTCTCCTCTTGAGTAAAAGGATAGCTAGCGGCCAGGAGAAATACAGTG
                          TACACTTCAGCTTCACTGACTTCTGGATTCTCCTCTTGAGTAAAAGGATAGCTAGCGGCCAGGAGAAATACAGTG
                             ACTTCAGCTTCACTGACTTCTGGATTCTCCTCTTGAGTAAAAGGATAGCTAGCGGCCAGGAGAAATACAGTGGAA
                              CTTCAGCTTCACTGACTTCTGGATTCTCCTCTTGAGTAAAAGGATAGCTAGCGGCCAGGAGAAATACAGTGGAAA
                               TTCAGCTTCACTGACTTCTGGATTCTCCTCTTGAGTAAAAGGATAGCTAGCGGCCAGGAGAAATACAGTGGAAAA
                                   GCTTCACTGACTTCTGGATTCTCCTCTTGAGTAAAAGGATAGCTAGCGGCCAGGATAAATACAGTGGAAAATGCA
                                         CTGACTTCTGGATTCTCCTCTTGAGTAAAAGGATAGCTAGCGGCCAGGAGAAATACAGTGGAAAATGCAAAACAA
                                              TTCTGGATTCTCCTCTTGAGTAAAAGGATAGCTAGCGGCCAGGAGAAATACAGTGGAAAATGCAAAACAACGAAA
                                               TCTGGATTCTCCTCTTGAGTAAAAGGATAGCTAGCGGCCAGGAGAAATACAGTGGAAAATGCAAAACAACGAAAT
                                               TCTGGATTCTCTTCTTGAGTAAAAGGATAGCTAGCGGCCAGGAGAAATACAGTGGAAAATGCAAAACAACGAAAT
                                                CTGGATTCTCCTCTTGAGTAAAAGGATAGCTAGCGGCCAGGAGAAATACAGTGGAAAATGCAAAACAACGAAATT
                                                NTGGATTCTCCTCTTGAGTAAAAGGATAGCTAGCGGCCAGGAGAAATACAGTGGAAAATGCAAAACAACGAAATT
                                                CTGGATTCTCCTCTTGAGTAAAAGGATAGCTAGTGGCCAGGAGAAATACAGTGGAAAATGCAAAACAACGAAATT
                                                CTGGATTTTCCTCTTGAGTAAAAGGATAGCTAGCGGCCAGGAGAAATACAGTGGAAAATGCAAAACAACGAAATT
                                                CTGGATTCTCCTCTTGAGGAAACGGATAGCTAGCGGCCAGGAGAAATACAGTGGAAAATGCAAAACAACGAAATT
                                                CTGGATTCTCCTCTGGAGTAAAAGGATAGCTAGCGGCCAGGAGAAATACAGTGGAAAATGCAAAACAACGAAATT
                                                CTGGATTCTCCTCTTGAGAAAAAGGATAGCTAGCGGCCAGGAGAAATACAGTGGAAAATGCAAAACAACGAAATT
                                                 TGGATTCTCCTCTTGAGTAAAAGGATAGCTAGCGGCCAGGAGAAATACAGTGGAAAATGCAAAACAACGAAATTA
                                                  GGATTCTCCTCTTGAGTAAAAGGATAGCTAGCGGCCAGGAGAAATACAGTGGAAAATGCAAAACAACGAAATTAT
                                                  TGATTCTCCTCTTGAGTAAAAGGATAGCTAGCGGCCAGGAGAAATACAGTGGAAAATGCAAAACAACGAAATTAT
                                                  GGATTTTCCTCTTGAGTAAAAGGATAGCTAGCGGCCAGGAGAAATACAGTGGAAAATGCAAAACAACGAAATTAT
                                                  GGATTTTCCTCTTGAGTAAAAGGATAGCTAGCGGCCAGGAGCAATACAGTGGAAAATGCAAAACAACGAAATTAT
                                                     TTCTCCTCTTGAGTAAAAGGATAGCTAGCGGCCAGGAGAAATACAGTGGAAAATGCAAAACAACGAAATTATAAA
                                                       CTCCTCTTGAGTAAAAGGATAGCTAGCGGCCAGGAGAAATACAGTGGAAAATGCAAAACAACGAAATTATAAAGC
                                                       CTTCTCTTGAGTAAAAGGATAGCTAGCGGCCAGGAGAAATACAGTGGAAAATGCAAAACAACGAAATTATAAAGC
                                                       CTCCTCTTGAGTAAAAGGATAGCTAGCGGCCAGGAGAAATACATTGGAAAATCCAAAACAACGAAATTATAAAGC
                                                       CTCCTCTTGAGTAAAAGGATAGCTAGCGGCCTGGAGAAATACAGTGGAAAATGCAAAACAACGAAATTATAAAGC
                                                         CCTCTTGAGTAAAAGGATAGCTAGCGGCCAGGAGAAATACAGTGGAAAATGCAAAACAACGAAATTATAAAGCCT
                                                          CTCTTGAGTAAAAGGATAGCTAGCGGCCAGGAGAAATACAGTGGAAAATGCAAAACAACGAAATTATAAAGCCTG
                                                           TCTTGAGTAAAAGGATAGCTAGCGGCCAGGAGAAATACAGTGGAAAATGCAAAACAACGAAATTATAAAGCCTGC
                                                            CTTGAGTAAAAGGATAGCTAGCGGCCAGGAGAAATACAGTGGAAAATGCAAAACAACGAAATTATAAAGCCTGCC
                                                            CTTGAGTAAAAGGATAGCTAGCGGCCAGGAGGAATACAGTGGAAAATGCAAAACAACGAAATTATAAAGCCTGCC
                                                            CTTGAGTAAAAGGATAGCTAGCGGTCAGGAGAAATACAGTGGAAAATGCAAAACAACGAAATTATAAAGCCTGCC
                                                             TTGAGTAAAAGGATAGCTAGCGGCCAGGAGAAATACAGTGGAAAATGCAAAACAACGAAATTATAAAGCCTGCCA
                                                               GAGTAAAAGGATAGCTAGCGGCCAGGAGAAATACAGTGGAAAATGCAAAACAACGAAATTATAAAGCCTGCCAAA
```
