# Supplementary material for: Recurrent rearrangements of the Myb/SANT-like DNA-binding domain containing 3 gene (MSANTD3) in salivary gland acinic cell carcinoma
Source: PLoS One. 2017 Feb 17;12(2):e0171265. doi: 10.1371/journal.pone.0171265 (PMC5315303; doi:10.1371/journal.pone.0171265)
Supplement: S2 Fig — Shown are the 78 sequencing reads spanning the predicted PRB3-ZNF217 junction, identified from the Chimeriscan analysis. (PDF) [file pone.0171265.s002.pdf]

S2 Fig. *PRB3*-*ZNF217* junction-spanning reads

```
  ←---------------------------PRB3 exon 1/2-----------------------------||------------------------ZNF217 exon 2-------------------------------→
5'-CTGGCCCTGAGCTCAGCTCAGAGCTTAAATGAAGATGTCAGCCAGGAAGAATCTCCCTCCGTAATATCAGGGTTTGGAAATCCCTTGTCTCCAGGTTGCTGGGATTGACTTCTTGCTCAATTGAAACACTCATTCAATGG-3'

Fusion junction-spanning reads:
   CTGGCCCTGAGCTCAGCTCAGAGCTTAAATGAAGATGTCAGCCAGGAAGAATCTCCCTCCGTAATATCAGGGTTT
   CTGGCCCTGAGCTCAGCTCAGGGCTTAAATGAAGATGTCAGCCAGGAAGAATCTCCCTCCGTAATATCAGGGTTT
   CTGGCCCTGAGCTCAGCTCAGAGCTTAAATGAAGAGGTCAGCCAGGAAGAATCTCCCTCCGTAATATCAGGGTTT
      GCCCTGAGCTCAGCTCAGAGCTTAAATGAAGATGTCAGCCAGGAAGAATCTCCCTCCGTAATATCAGGGTTTGGA
       CCCTGAGCTCAGCTCAGAGCTTAAATGAAGATGTCAGCCAGGAAGAATCTCCCTCCGTAATATCAGGGTTTGGAA
        CCTGAGCTCAGCTCAGAGCTTAAATGAAGATGTCAGCCAGGAAGAATCTCTCTCCGTAATATCAGGGTTTGGAAA
        CCTNAGCTCAGCTCAGAGCTTAAATGAAGATGTCAGCCAGGAAGAATCTCTCTCCGTAATATCAGGGTTTGGAAA
        CCTGAGCTCAGCTCAGAGCTTAAATGAAGATGTCAGCCAGGAAGAATCTCCCTCCGTAATATCAGGGTTTGGAAA
         CTGAGCTCAGCTCAGAGCTTAAATGAAGATGTCAGCCAGGAAGAATCTCCCTCCGTAATATCAGGGTTTGGAAAT
         CTGAGCTCAGCTCAGAGCTTAAATGAAGATGTCAGCCAGGAAGAATCTCCCTCCGTAATATCAGGGTTTGGAAGT
         NTGAGCTCAGCTCAGAGCTTAAATGAAGATGTCAGCCAGGAAGAATCTCCCTCCGTAATATCAGGGTTTGGAAAT
              CTCAGCTCAGAGCTTAAATGAAGATGTCAGCCAGGAAGAATCTCCCTCCGTAATATCAGGGTTTGGAAATCCCTT
                           TTAAATGAAGATGTCAGCCAGGAAGAATCTCCCTCCGTAATATCAGGGTTTGGAAATCCCTTGTCTCCAGGTTGC
                           TTAAATGAAGATGTCAGCCAGGAAGACTCTCCCTCCGTAATATCAGGGTTTGGAAATCCCTTGTCTCCAGGTTGC
                           TTAAATGAAGATGTCAGCCAGGAAGAATCTCCCTCCGTAATATCCGGGTTTGGAATTCCCTTGTCTCCAGGTTGC
                            TAAATGAAGATGTCAGCCAGGAAGAATCTCCCTCCGTAATATCAGGGTTTGGAAATCCCTTGTCTCCAGGTTGCT
                            TAAATGAACATGTCAGCCAGGAAGAATCTCCCTCCGTAATATCAGGGTTTGGAAATCCCTTGTCTCCAGGTTGCT
                             AAATGAAGATGTTAGCCAGGAAGAATCTCCCTCCGTAATATCAGGGTTTGGAAATCCCTTGTCTCCAGGTTGCTG
                             AAATGAAGATGTCAGCCAGGAAGAATCTCCCTCCGTAATATCAGGGTTTGGAAATCCCTTGTCTCCAGGTTGCTG
                              AATGAAGATGTCAGCCAGGAAGAATCTCCCTCCGTAATATCAGGGTTTGGAAATCCCTTGTCTCCAGGTTGCTGG
                              AATCAAGATGTCAGCCAGGAAGAATCTCCCTGCGTAATATCAGGGTTTGGAAATCCCTTGTCTCCAGGTTGCTGG
                              AATGAAGATGTCAGCCAGGAAGAATCTCCCTCCGTAATATCAGGGTTTGGAAATCCCTTGTCTCCAGGTTGCGGG
                               ATGAAGATGTCAGCCAGGAAGAATCTCCCTCCGTAATATCAGGGTTTGGAAATCCCTTGTCTCCAGGTTGCTGGG
                               ATGAAGATGTCAGCCAGGAAGAATCTCCCTCCGTAATATCAGGGTTTGGAAATCCCTTGTCTCCAGGTTGTTGGG
                                TGAAGATGTCAGCCAGGAAGAATCTCCCTCCGTAATATCAGGGTTTGGAAATCCCTTGTCTCCAGGTTGCTGGGA
                                 GAAGATGTCAGCCAGGAAGAATCTCCCTCCGTAATATCAGGGTTTGGAAATCCCTTGTCTCCAGGTTGCTGGGAT
                                     ATGTCAGCCAGGAAGAATCTCCCTCCGTAATATCAGGGTTTGGAAATCCCTTGTCTCCAGGTTGCTGGGATTGAC
                                     ATGTCAGCCAGGAAGAATCTCCCTCCGTAATATTAGGGTTTGGAAATCCCTTGTCTCCAGGTTGCTGGGATTGAC
                                      TGTCAGCCAGGAAGAATCTCCCTCCGTAATATCAGGGTTTGGAAATCCCTTGTCTCCAGGTTGCTGGGATTGACT
                                       GTCAGCCAGGAAGAATCTCCCTCCGTAATATTAGGGTTTGGAAATCCCTTGTCTCCAGGTTGCTGGGATTGACTT
                                       GTCAGCCAGGAAGAATCTCCCTCCGTAATATCAGGGTTTGGAAATCCCTTGTCTCCAGGTTGCTGGGATTGACTT
                                       GTCAGCCAGGAAGAATCTCCCTCCGTAATATCAGGGGTTGGAAATCCCTTGTCTCCAGGTTGCTGGGATTGACTT
                                          AGCCAGGAAGAATCTCCCTCCGTAATATCAGGGTTTGGAAATCCCTTGTCTCCAGGTTGCTGGGATTGACTTCTT
                                           GCCAGGAAGAATCTCCCTCCGTAATATCAGGGTTTGGAAATCCCTTGTCTCCAGGTTGCTGGGATTGACTTCTTG
                                           NCCAGGAAGAATCTCCCTCCGTAATATCAGGGTTTGGAAATCCCTTGTCTCCAGGTTGCTGGGATTGACTTCTTG
                                           GCCAGGAAGAATCTCCCTCCGTAATATCAGGGTTAGGAAATCCCTTGTCTCCAGGTTGCTGGGATTGACTTCTTG
                                             CAGGAAGAATCTCCCTCCGTAATATCAGGGTTTGGAAATCCCTTGTCTCCAGGTTGCTGGGATTGACTTCTTGCT
                                               GGAAGAATCTCCCTCCGTAATATCAGGGTTTGGAAATCCCTTGTCTCCAGGTTGCTGGGATTGACTTCTTGCTCA
                                               GGTAGAATCTCCCTCCGTAATATCAGGGTTTGGAAATCCCTTGTCTCCAGGTTGCTGGGATTGACTTCTTGCTCA
                                               GGAAGAATCTCCCTCCGTAATATCAGGGTTTGGAAATCCCTTGTCTCTAGGTTGCTGGGATTGACTTCTTGCTCA
                                                GAAGAATCTCTCTCCGTAATATCAGGGTTTGGAAATCCCTTGTCTCCAGGTTGCTGGGATTGACTTCTTGCTCAA
                                                GAAGAATCTCCCTCCGTAATATCAGGGTTTGGAAATCCCTTGTCTCCAGGTTGCTGGGATTGACTTCTTGCTCAA
                                                 AAGAATCTCCCTCCGTAATATCAGGGTTTGGAAATCCCTTGTCTCCAGGTTGCTGGGATTGACTTCTTGCTCAAT
                                                  AGAATCTCCCTCCGTAATATCAGGGTTTGGAAATCCCTTGTCTCCAGGTTGCTGGGATTGACTTCTTGCTCAATT
                                                  AGAATCTCCCTCCGTAATATCAGGGTTTGGAAATCCCTTGTCTCCAGGTTGCTGGGATTTACTTCTTGCTCAATT
                                                  AGAATCTCCCTCCGTAATATCAGGGTTTGGAAATTCCTTGTCTCCAGGTTGCTGGGATTGACTTCTTGCTCAATT
                                                   GAATCTCCCTCCGTAATATCAGGGTTTGGAAATCCCTTGTCTCCAGGTTGCTGGGATTGACTTCTTGCTCAATTG
                                                   GAATCTCCCTCCGTAATATCAGGGTTTGGAAATCCCTTGTCTCCAGGTTGCTGGGATTGACTTCTTGNTCAATTG
                                                   GAATCTCCCTCCGTAATATCAGGGTTTGGAAATCCCTTGTCTCCAGGTTGCTGGGATTGACTTCTTGCTCAACTG
                                                   GAATCTCCCTCCGTAATATCAGGGTTTGGAAATCCCTTGTCTCCAGGTTGCCGGGATTGACTTCTTGCTCAATTG
                                                   CAATCTCCCTCCGTAATATCAGGGTTTGGAAATCCCTTGTCTCCAGGTTGCTGGGATTGACTTCTTGCTCAATTG
                                                    AATCTCCCTCCGTAATATCAGGGTTTGGAAATCCCTTGTCTCCAGGTTGCTGGGATTGACTTCTTGCTCAATTGA
                                                      TCTCCCTCCGTAATATCAGGGTTTGGAAATCCCTTGTCTCCAGGTTGCTGGGATTGACTTCTTGCTCAATTGAAA
                                                      TCTCCCTCCGTAATATCAGGGTTTGGAAATCCCTTGTCTCCAGGTTGCTGGGATTGACTTCTTGCTCAATTGAAN
                                                      TCTCCCTCCGTAATATCAGGGTTTGGAAATCCCTTGTCTCCAGGTTGCTGGGATTGACATCTTGCTCAATTGAAA
                                                      TCTCCCTCCGTAATATCAGGGTTTGGAAATCCCTTGTCTCTAGGTTGCTGGGATTGACTTCTTGCTCAATTGAAA
                                                       CTCCCTCCGTAATATCAGGGTTTGGAAATCCCTTGTCTCCAGGTTGCTGGGATTGACTTCTTGCTCAATTGAAAC
                                                          CCTCCGTAATATCAGGGTTTGGAAATCCCTTGTCTCCAGGTTGCTGGGATTGACTTCTTGCTCAATTGAAACACT
                                                          CCTCCGTAATATCAGGGTTTGGAAATCCCTTGTCTCCAGGTTGCTGGGATTGACTTCTTGCTCAATTGAAACACA
                                                           CTCCGTAATATCAGGGTTTGGAAATCCCTTGTCTCCAGGTTGCTGGGATTGACTTCTTGCTCAATTGAAACACTC
                                                           CTCCGTAATATCAGGGTTTGGAAATCCCTTGTCTCCAGGTTGCTGGGATTGACTTCTTGCTCAATTGAAATACTC
                                                           CTCCGTCATATCAGGGTTTGGAAATCCCTTGTCTCCAGGTTGCTGGGATTGACTTCTTGCTCAATTGAAACACTC
                                                           CTCCGTAATATCAGGGTTTGGAAATCCCTTGTCTCCAGGTTGCTGGGATTGACATCTTGCTCAATTGAAACACTC
                                                           CTCCGTAATATCAGGGTTTGGAAATCCCTTGTCTCCAGGTTGCTGGGATTGACTTCTTGCTCAATTGAAATACGC
                                                           CTCCGTAATATCAGGGTTTGGAAATCCCTAGTCTCCAGGTTGCTGGGATTGACTTCTTGCTCAATTGAAACACCC
                                                           CTCCGTAATATCAGGGTTTGGAAATCCCTTGTCTCCAGGTTGCTGGGATTGACTTCTTGCTCAATTGAAACACCC
                                                            TCCGTAATATCAGGGTTTGGAAATCCCTTGTCTCCAGGTTGCTGGGATTGACTTCTTGCTCAATTGAAACACTCA
                                                            TCCGTAATATCAGGGTTTGGAAATCCCTTGTCTCCAGGTTGCTGGGATTGACTTCTTGCTCAATTGAACCACTCA
                                                            TCCGTAATATCAGGGTTTGGAAATCCCTTGTCTCCAGGTTGCTGGGATTGCCTTCTTGCTCAATTGAAACACTCA
                                                            TCCGTAATATCAGGGTTTGGAAATCCCTTGTCTCCAGGTTGCTGGGATTGACTTCTTGCTCAATTGAAACACTCG
                                                              CGTAATATCAGGGTTTGGAAATCCCTTGTCTCCAGGTTGCTGGGATTGACTTCTTGCTCAATTGAAACACTCATT
                                                               GTAATATCAGGGTTTGGAAATCCCTTGTCTCCAGGTTGCTGGGATTGACTTCTTGCTCAATTGAAACACTCATTC
                                                                TAATATCAGGGTTTGGAAATCCCTTGTCTCCAGGTTGCTGGGATTGACTTCTTGCTCAATTGAAACACTCATTCA
                                                                  ATATCAGGGTTTGGAAATCCCTTGTCTCCAGGTTGCTGGGATTGACTTCTTGCTCAATTGAAACACTCATTCAAT
                                                                   TATCAGGGTTTGGAAATCCCTTGTCTCCAGGTTGCTGGGATTGACTTCTTGCTCAATTGAAACACTCATTCAATG
                                                                    ATCAGGGTTTGGAAATCCCTTGTCTCCAGGTTGCTGGGATTGACTTCTTGCTCAATTGAAACACTCATTCAATGG
                                                                    ATCAGGGTTTGGAAATCCCTTGTCTCCAGGTTGCTGGGATTGACCTCTTGCTCAATTGAAACACTCATTCAATGG
                                                                    ATCAGGGTTTGGAAATCCCTTGTCTCCAGGTTGCTGGGATGGACTTCTTGCTCAATTGAAACACTCATTCAATGG
```
