# Supplementary material for: Recurrent rearrangements of the Myb/SANT-like DNA-binding domain containing 3 gene (MSANTD3) in salivary gland acinic cell carcinoma
Source: PLoS One. 2017 Feb 17;12(2):e0171265. doi: 10.1371/journal.pone.0171265 (PMC5315303; doi:10.1371/journal.pone.0171265)

S3 Fig

Higher levels in cancer:

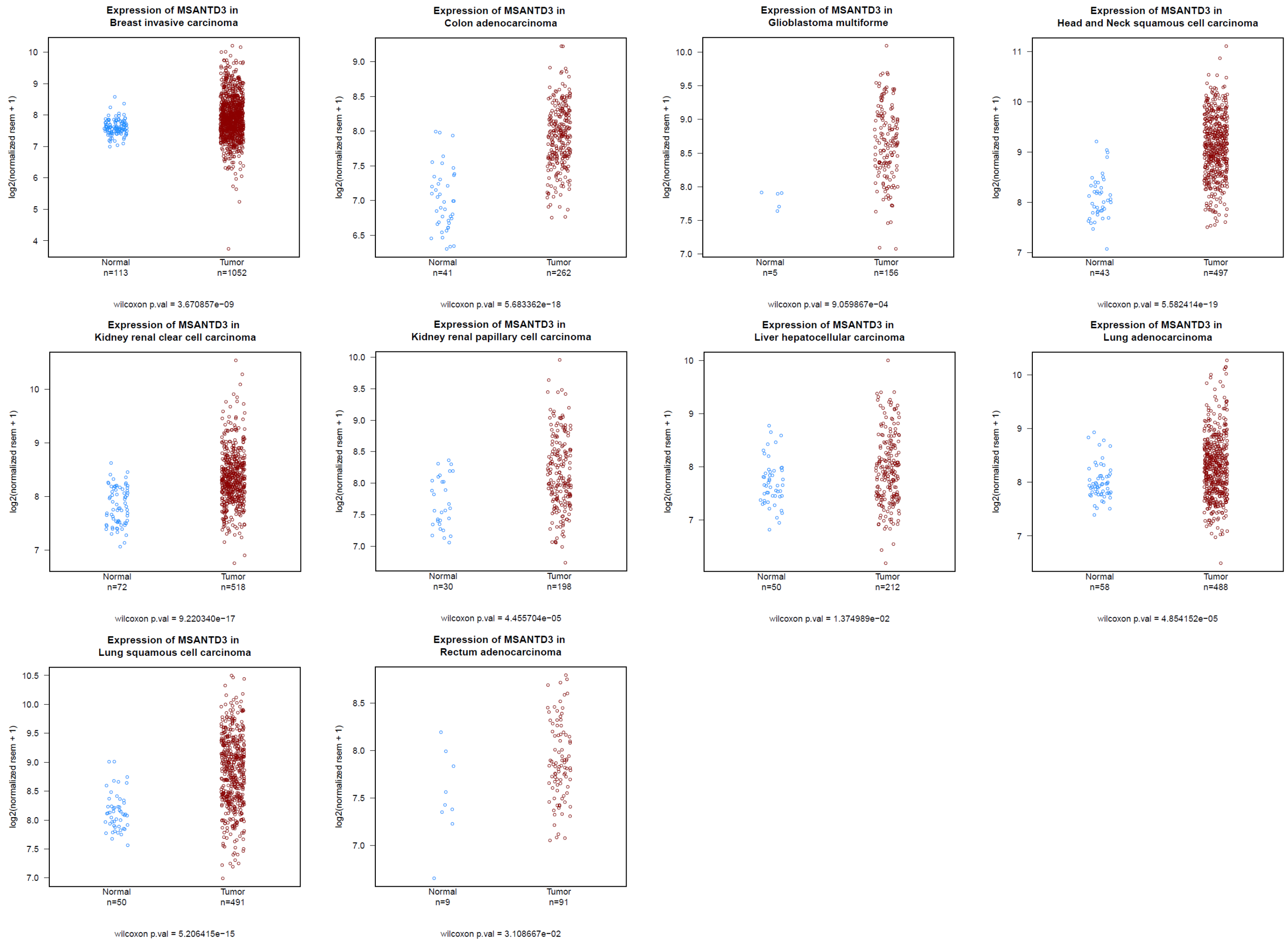

No significant difference (tumor *vs*. normal):

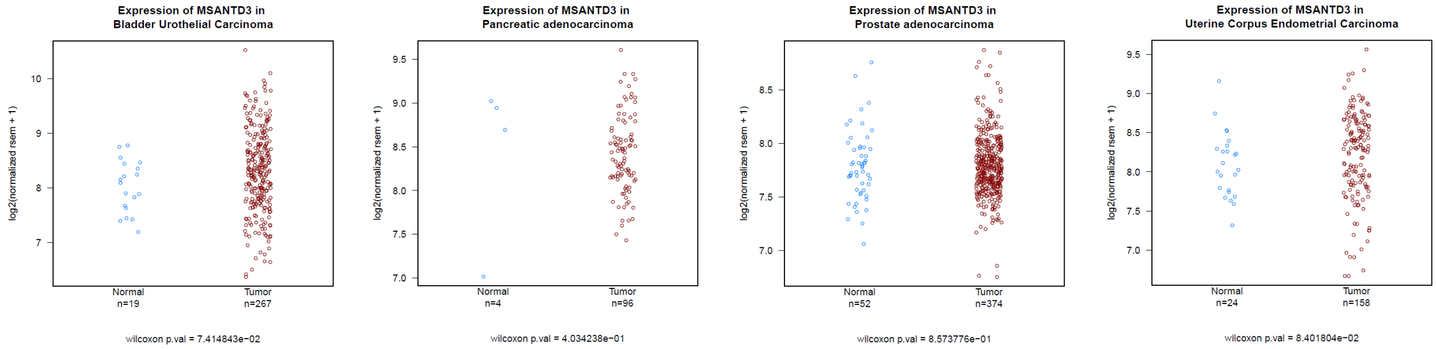

Higher levels in normal:

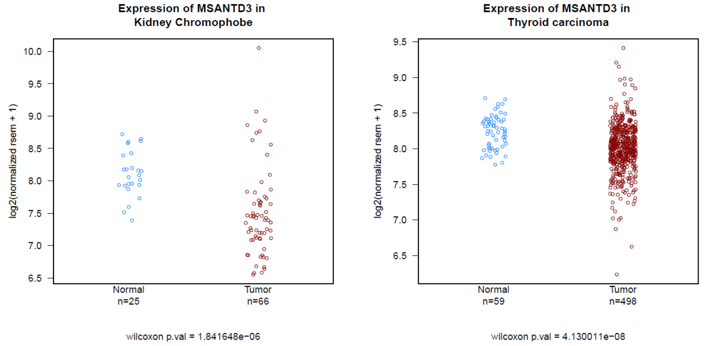

Supplement: S3 Fig — Plots display normalized transcript levels for cancer vs. normal samples, shown for those TCGA cancer types where there are sufficient sample numbers to perform a statistical analysis (tumor vs. normal; Wilcoxon P-value indicated). Plots assembled using Wanderer (see Methods). (PDF) [file pone.0171265.s003.pdf]
