# Supplementary figures and images for: Recurrent rearrangements of the Myb/SANT-like DNA-binding domain containing 3 gene (MSANTD3) in salivary gland acinic cell carcinoma
Source: PLoS One. 2017 Feb 17;12(2):e0171265. doi: 10.1371/journal.pone.0171265 (PMC5315303; doi:10.1371/journal.pone.0171265)

S4 Fig

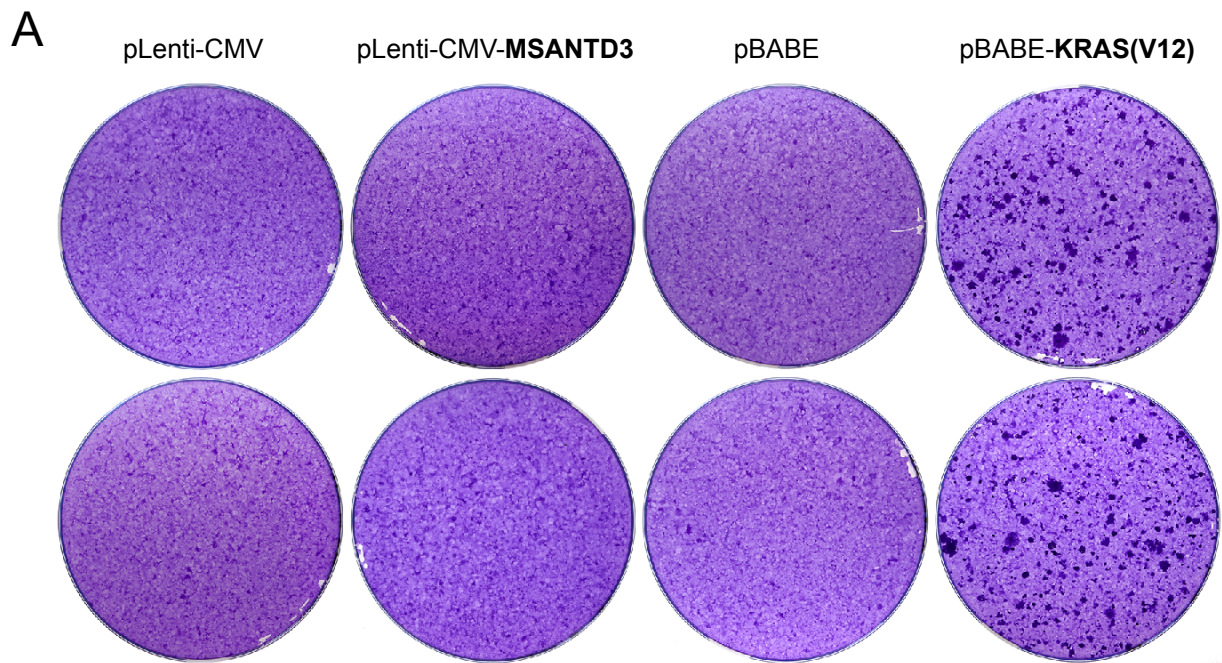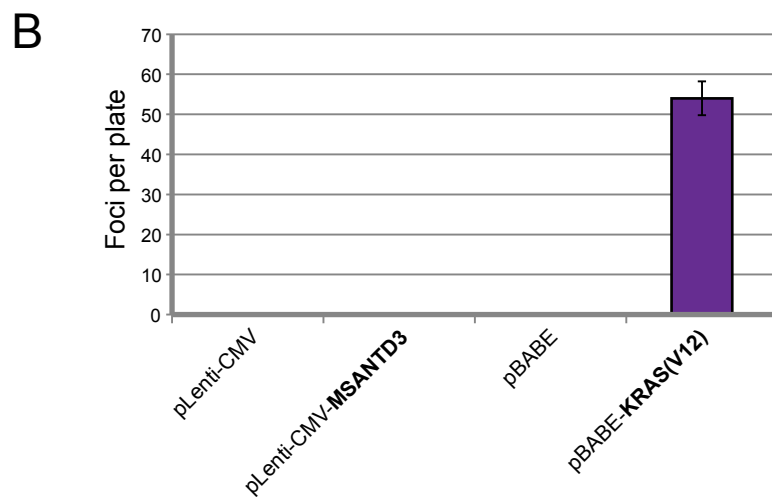

Supplement: S4 Fig — (A) Focus formation assay in NIH-3T3 cells transduced with pLenti-CMV-MSANTD3 (vs. pLenti-CMV empty vector control), or positive control pBABE-KRAS(V12) (vs. pBABE empty vector control). Crystal violet stained foci were manually counted in triplicate 10cm plates; representative plates shown. (B) Graphical display of counted foci. Note, no foci (>3mm) were observed following MSANTD3 overexpression. (PDF) [file pone.0171265.s004.pdf]
