# Supplementary material for: Recurrent rearrangements of the Myb/SANT-like DNA-binding domain containing 3 gene (MSANTD3) in salivary gland acinic cell carcinoma
Source: PLoS One. 2017 Feb 17;12(2):e0171265. doi: 10.1371/journal.pone.0171265 (PMC5315303; doi:10.1371/journal.pone.0171265)
Supplement: S1 Table — (PDF) [file pone.0171265.s005.pdf]

**S1 Table.** Canonical pathway gene sets found enriched by GSEA analysis

| **Enriched gene sets upregulated with MSANTD3 overexpression (FDR<0.25; top 10 only)** | **Size** | **NOM p-val** | **FDR q-val** |
|---|---|---|---|
| KEGG RIBOSOME | 75 | <0.001 | <0.00001 |
| REACTOME PEPTIDE CHAIN ELONGATION | 75 | <0.001 | <0.00001 |
| REACTOME 3 UTR MEDIATED TRANSLATIONAL REGULATION | 81 | <0.001 | <0.00001 |
| REACTOME NONSENSE MEDIATED DECAY ENHANCED BY THE EXON JUNCTION COMPLEX | 87 | <0.001 | <0.00001 |
| REACTOME INFLUENZA VIRAL RNA TRANSCRIPTION AND REPLICATION | 87 | <0.001 | <0.00001 |
| REACTOME SRP DEPENDENT COTRANSLATIONAL PROTEIN TARGETING TO MEMBRANE | 94 | <0.001 | <0.00001 |
| REACTOME FORMATION OF THE TERNARY COMPLEX AND SUBSEQUENTLY THE 43S COMPLEX | 32 | <0.001 | <0.00001 |
| REACTOME INFLUENZA LIFE CYCLE | 115 | <0.001 | <0.00001 |
| REACTOME TRANSLATION | 116 | <0.001 | <0.00001 |
| REACTOME ACTIVATION OF THE MRNA UPON BINDING OF THE CAP BINDING COMPLEX AND EIFS AND SUBSEQUENT BINDING TO 43S | 38 | <0.001 | <0.00001 |
| | | | |
| **Enriched gene sets downregulated with MSANTD3 overexpression (FDR<25%)** | **Size** | **NOM p-val** | **FDR q-val** |
| KEGG ADHERENS JUNCTION | 48 | <0.001 | 0.009 |
| ST G ALPHA I PATHWAY | 18 | <0.001 | 0.07 |
